# Clinical course and implications of congenital nasal pyriform stenosis and solitary median maxillary central incisor in a newborn: a case report

**DOI:** 10.1186/1752-1947-8-215

**Published:** 2014-06-20

**Authors:** Lutz Günther, Aynur Sari-Rieger, Karsten Jablonka, Jan Rustemeyer

**Affiliations:** 1 Department of Oral and Maxillofacial Surgery, Plastic Operations, Klinikum Bremen-Mitte, School of Medicine of the University of Göttingen, St Jürgen Strasse 1, 28177 Bremen, Germany; 2 Department of Radiology, Paediatric Radiology, Klinikum Bremen-Mitte, School of Medicine of the University of Göttingen, St Jürgen Strasse 1, 28177 Bremen, Germany

**Keywords:** Congenital nasal pyriform aperture stenosis, Solitary median maxillary central incisor, Malformations, Associated anomalies

## Abstract

**Introduction:**

Congenital nasal pyriform aperture stenosis and solitary median maxillary central incisor are uncommon anomalies and are associated with further malformations. Solitary median maxillary central incisor itself has initially no impact on a child’s health, but congenital nasal pyriform aperture stenosis is a potentially life-threatening condition.

**Case presentation:**

A Caucasian baby boy showed severe dyspnoea and was intubated orotracheally. Multiple anomalies were detected, including urogenital and craniofacial malformations. Computed tomography scans revealed congenital nasal pyriform aperture stenosis with a diameter of 4.9mm and a solitary median maxillary central incisor. A 3.0mm tube was inserted in his left nasal cavity, and the baby was able to breathe sufficiently and spontaneously. The nasal tube was removed after seven days, and the baby was discharged under application of decongestant drops. After seven months, the baby was readmitted with respiratory distress, and surgery was carried out using an intraoral sublabial approach. The stenotic area of the pyriform aperture was widened, and 3.0mm tubes were inserted in both nasal cavities for 10 days. Over a period of six months, no further respiratory distress has occurred.

**Conclusions:**

The decision to perform surgery was delayed since the baby’s nasal breathing was adequate as a result of the insertion of a nasal tube. Since treatment depends on the severity of symptoms, it is appropriate in some cases to take a conservative approach at first, and to keep surgery as a last resort. Once a conservative approach has been selected for congenital nasal pyriform aperture stenosis, awareness of the life-threatening nature of the condition should be kept in mind, and a surgical approach must still be taken into account.

## Introduction

Congenital nasal pyriform aperture stenosis (CNPAS) is an unusual neonatal upper airway obstruction, which was first described in 1989 [1]. The true incidence is still unknown. It is estimated that CNPAS occurs at a frequency of about one-fourth to one-third of choanal atresia, which in turn has a reported incidence of 1 in 5,000 to 8,000 live births [2,3]. CNPAS can lead to life-threatening conditions since newborns are obligate nasal breathers in the first weeks of their life. Hence, early diagnosis is vital for the appropriate management of the condition, which could be either conservative or surgical, and depends on the severity of symptoms. Associated malformations include hypopituitarism, and craniofacial, cardiac and urogenital anomalies [4]. Furthermore, studies have confirmed the suggestion that CNPAS even represents a manifestation of holoprosencephaly (HPE) [5]. A solitary median maxillary central incisor (SMMCI) can be detected in up to 60 percent of CNPAS cases. SMMCI is a rare dental anomaly that has been considered to be a part of a complex disorder consisting of multiple, mainly midline defects of development. It results from unknown factors operating *in utero* about the 35th to the 38th day from conception and is estimated to occur in 1 in 50,000 live births. SMMCI can be found in patients with specific chromosomal abnormalities, including missense mutation in the *Sonic Hedgehog (SHH)* gene (*I111F*) at 7q36. In accordance with CNPAS, SMMCI may include microforms and the full spectrum of HPE as well [6-8].

In this case report, we present the clinical course of a newborn with CNPAS and SMMCI, and discuss the management options. Our experience may contribute to further interdisciplinary decision-making for these rare conditions.

## Case presentation

**Figure 1:**
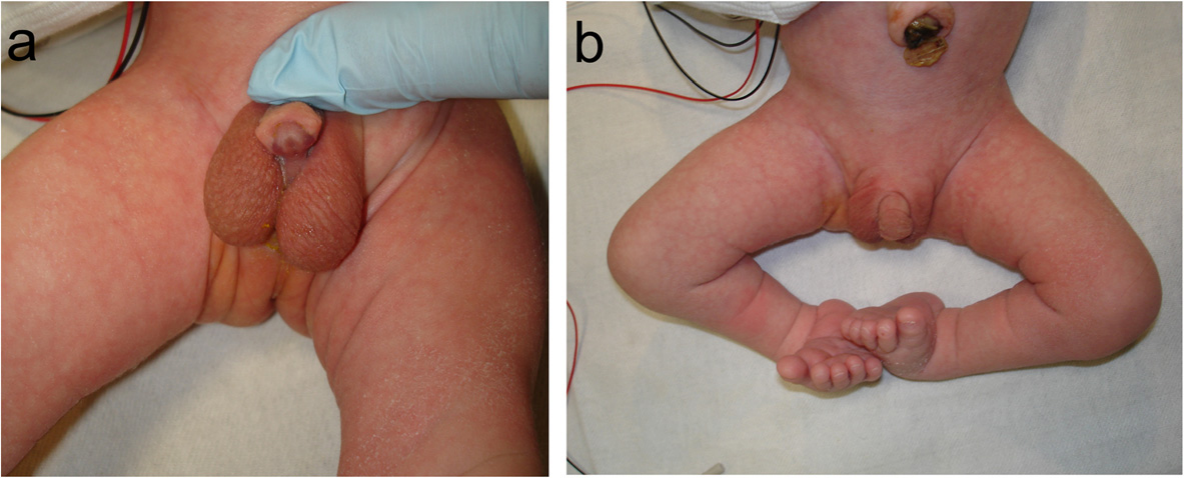
**Associated anomalies.** Multiple anomalies including penile hypospadia and scrotum bipartitum **(a)**, clubfeet and umbilical hernia **(b)**.

**Figure 2:**
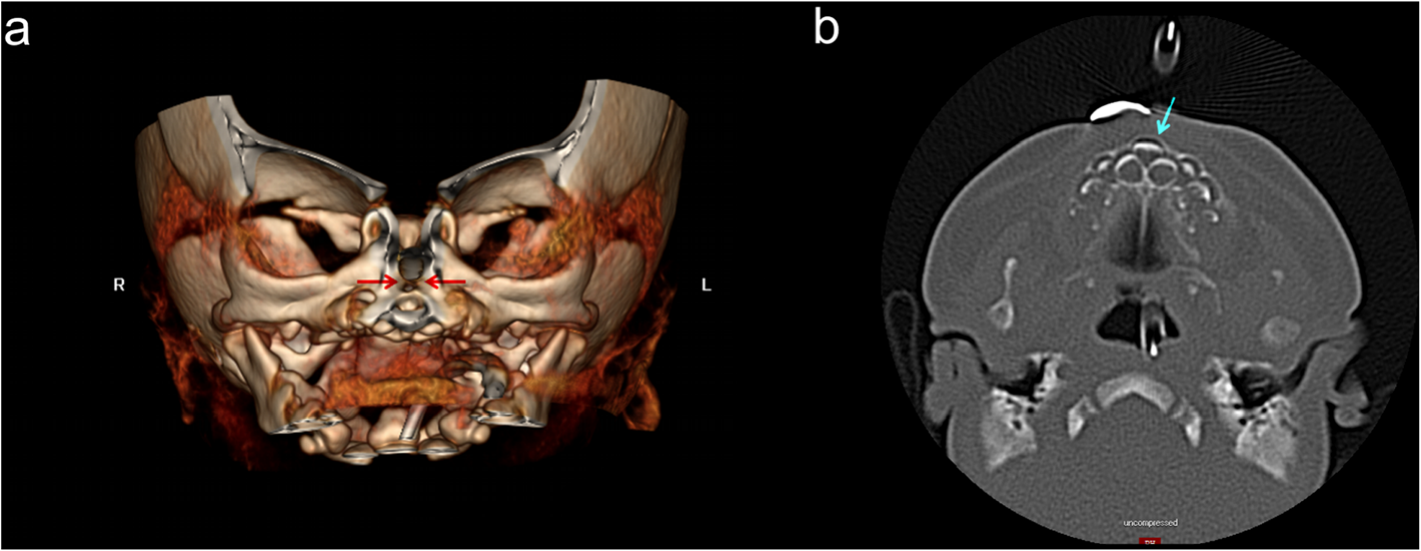
**Computed tomography scans showing congenital nasal pyriform aperture stenosis and solitary median maxillary central incisor.** A three-dimensional reconstructed computed tomography scan shows the amount of congenital nasal pyriform aperture stenosis (arrows). R, right; L, left. A maximum diameter of only 4.9mm is apparent **(a)**. A computed tomography scan reveals a solitary median maxillary central incisor (arrow) precisely in the midline of the maxillary dental arch **(b)**.

**Figure 3:**
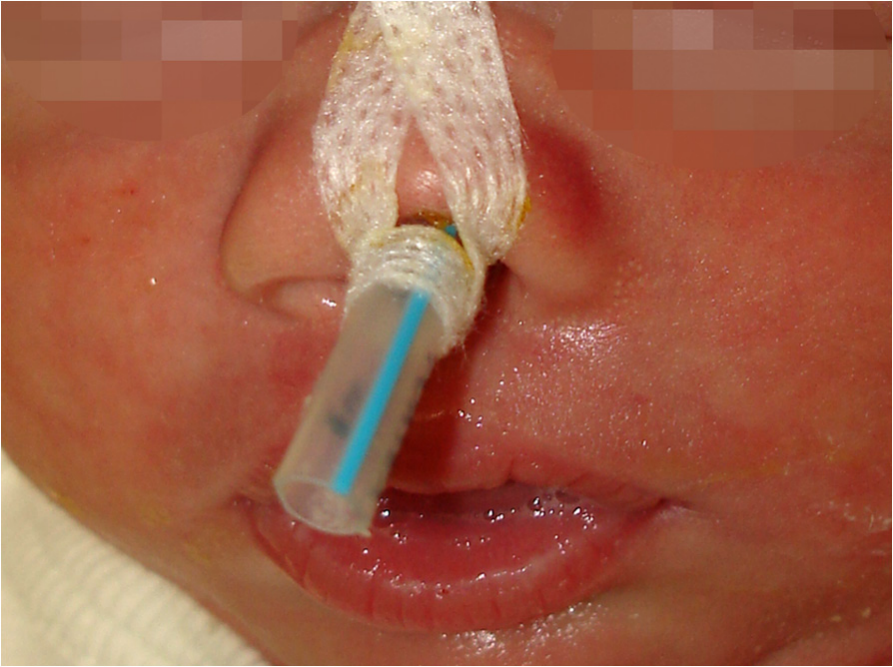
**Incorporated 3.00mm tube.** A 3.0mm tube was incorporated in the left nasal cavity to achieve sufficient breathing.

**Figure 4:**
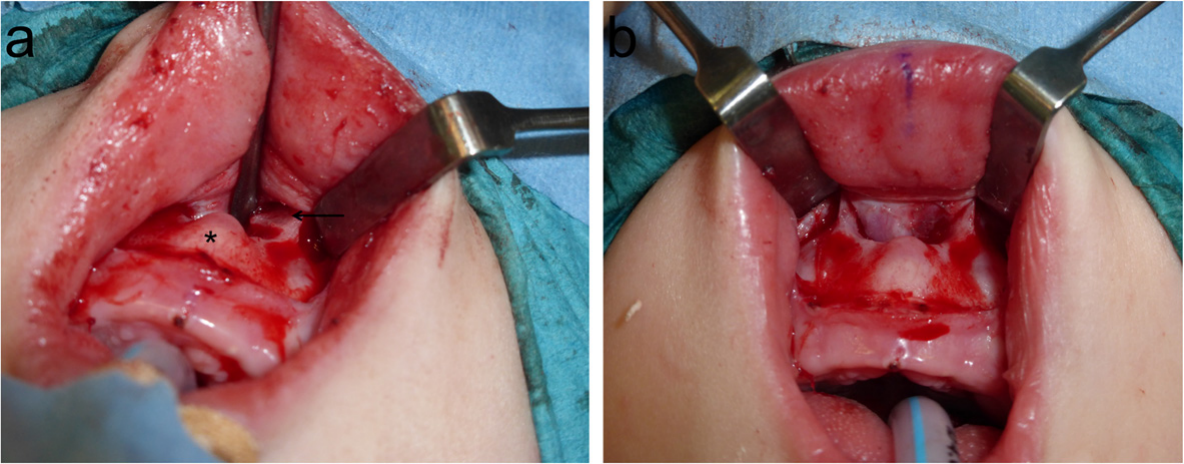
**Intraoperative findings.** Prominent stenosis of the pyriform aperture (arrow) and protrusion of the median alveolus with an underlying solitary median maxillary central incisor (asterisk) **(a)**. Situs after widening the pyriform aperture **(b)**.

A Caucasian baby boy born full term at 40 weeks, with a body weight of 2.88kg, height of 47cm and cranial circumference of 36cm, had Apgar scores of 7, 8 and 9 at 1, 5, and 10 minutes after birth, respectively. The newborn showed symptoms of respiratory distress and stridor, and was consequently orotracheally intubated for continuous positive airway pressure (CPAP) ventilation. Multiple anomalies were detected through performing a clinical examination and ultrasonic scans, including an ostium secundum defect, penile hypospadia, scrotum bipartitum, clubfeet and umbilical hernia (Figure 1). In addition, his nose appeared hypoplastic, and his nostrils were anteverted. A cranial computed tomography (CT) scan revealed stenosis of the pyriform aperture with a maximum diameter of 4.9mm and an SMMCI (Figure 2). Further intra- or extracranial anomalies, including HPE or a choanal stenosis, could be ruled out. Three days later, after the baby had undergone herniotomy by pediatric surgeons, CPAP was ceased since the baby was able to breathe sufficiently spontaneously with a 3.0mm (inner diameter) tube incorporated in his left nasal cavity (Figure 3). Right-sided nasal passage and endoscopic rhinoscopy were not possible because of the extent of the anterior nasal stenosis. The nasal tube was removed after seven days and the baby was discharged without further respiratory distress. Topical decongestant drops were applied three times a day to enhance further extension of the nasal airway for 14 days. In addition, a setup for measuring oxygen saturation was installed at home. Shortly after discharge, redressement therapy for his clubfeet was initiated. Follow-up consultations every four weeks revealed continuing nasal obstruction, but sufficient breathing overall.Seven months after birth, the baby was readmitted to acute care with multiple episodes of dyspnea, inspiratory stridor and problems with nutrition, causing a dystrophic general condition. Only mouth breathing was possible, breaks for breathing were necessary during food intake and lying in the supine position led to a falling back of the tongue with cyanosis. Because of the worsening of his symptoms, and because a further attempt of conservative treatment with installation of nasal tubes failed, the baby underwent surgery one day after readmission. Through an intraoral sublabial approach, that provided for a midface degloving, the pyriform aperture could be reached easily and the double-sided stenotic area was widened by drilling and reshaping the bone with diamond burs, avoiding injuring the contiguous mucosal nasal soft tissue (Figure 4). Since intraoperative findings showed that the SMMCI did not contribute to the pyriform stenosis, no removal was carried out to spare maxillary bone integrity. After the operation, two silastic tubes with an inner diameter of 3.0mm and a length of 40mm were placed intranasally to stabilize the nasal airway. An attempt to install 3.5mm tubes was interrupted, since the small columnella immediately became cyanotic.

On postoperative day 3, orotracheal extubation was possible at the intensive care unit without complications. The nasal tubes were changed after five days and completely removed on postoperative day 10. Nasal decongestant drops were applied for another 14 days. Monitoring oxygen saturation by day and by night revealed no significant decreases even in the supine position, and nutrition without breathing breaks was possible. The baby was discharged and given appointments for close follow-up. Over a period of six months after surgery, the baby had no further symptoms of respiratory distress and was admitted to the pediatric surgery department for therapy for his other malformations.

## Discussion

In the present case report, it remains unclear whether CNPAS was accompanied by an SMMCI or *vice versa*. However, both malformations raise the possibility of further associated anomalies. Since neonates are obligatory nasal breathers, the possible life-threatening condition of a CNPAS needs immediate intervention after birth. Hence, the focus is primarily more on this malformation than on SMMCI. In general, management of CNPAS depends first on the overall prognosis of the patient, and second on the severity of obstruction. Patients with poor overall prognosis should be managed conservatively with an oropharyngeal airway. In patients with a good outlook, the choice of treatment is conservative for those with less severe obstruction, and surgical correction for those with complete obstruction [5]. The diagnosis of CNPAS is based on clinical evaluation, including nasal endoscopy and, especially, CT scans. The inability to pass a 5 French (F) catheter (outer diameter 1.67mm) and a radiographically measured pyriform opening less than 8 to 10mm in a full-term newborn are considered as a relevant stenosis. In turn, the aperture is considered satisfactory when it allows for the passage of an endotracheal tube stent with an inner diameter of 3.5mm [5,9].

In the present case, the decision for surgery was delayed for a considerable time since nasal breathing was adequate after initial intubation of the left nasal cavity, and later even without a nasal tube. As a result of growth, CNPAS became clinically apparent again at an age of seven months, and rendered surgery mandatory. However, since treatment depends on the severity of symptoms in borderline cases as presented in this report, it is appropriate to use the conservative therapeutic spectrum at first and keep surgery as the last resort. In general, surgical removal of the external bony margins of the pyriform aperture is successful regarding normal nasal breathing and pyriform aperture growth after surgery in further follow-up assessments. No bony restenosis of CNPAS with the necessity of a second operation is described in the literature [9,10]. However, reservation toward a primary surgical approach has been supported by results of a recent retrospective case note review. From a total of 10 identified cases of CNPAS, only 50 percent had to be managed surgically [4]. Accordingly, in another case series, 0.1 percent intranasal dexamethasone drops were used successfully for conservative treatment in three out of five patients [11]. Furthermore, possible complications of a surgical approach have to be evaluated, including columellar necrosis, which has been described in one out of 20 surgical cases, development of synechiae, and septal ulceration with septal perforation [6,10]. With respect to the reported complications following a surgical approach, in our case we discontinued the attempt to install larger tubes, since the small columnella became cyanotic. However, to reduce recurrence and scar-related stenosis after surgery, the use of nasal stents is recommended. To avoid septal complications and scarring after surgery, we changed nasal tubes after five days, and removed tubes not later than day 10, in accordance with the recommendations given in the literature in cases of isolated CNPAS [9]. Another reason for a delay in surgery in a considerable number of cases without a life-threatening condition is to gain valuable time until general anesthesia can be carried out. Of course, intraoperative hemorrhage within widening the pyriform aperture should not be comparable with hemorrhage within surgery on craniosynostosis. However, incidence of intraoperative hypoxemia increases with younger age, with the highest incidence in neonates, and is further complicated by syndrome-specific issues [12].

SMMCI was previously considered to be a simple midline defect of the dental lamina, but it is now recognized as a possible predictor of HPE of varying degrees in the proband, in members of the proband’s family, and in the family’s descendants [13]. Hence, genetic counseling is mandatory in these cases. The SMMCI tooth itself is a real central incisor tooth, although of unusual crown form, but not a supernumerary tooth like a mesiodens. It develops and erupts precisely in the midline of the maxillary dental arch. Only in very rare cases, does SMMCI contribute to the pyriform aperture stenosis. Otherwise, it is mainly an esthetic problem, managed by combined orthodontic, prosthodontic and oral surgical treatment. Alternatively, it can be left untreated [8].

## Conclusions

Once a conservative approach is selected for the treatment of CNPAS, awareness of the life-threatening nature of this malformation should be kept in mind. Since respiratory distress due to CNPAS can also become apparent during a child’s further growth, the immediate need for a surgical approach must still be taken into account. Therefore, close follow-up is highly recommended.

## Consent

Written informed consent was obtained from the parents of the patient for publication of this case report and any accompanying images. A copy of the written consent is available for review by the Editor-in-Chief of this journal.

## Competing interests

The authors declare that they have no competing interests.

## Authors’ contributions

LG, ASR, KJ and JR collected and analyzed patient data, administered therapy and reviewed the literature. KJ provided extra radiologic features including the three-dimensional computed tomography scans. JR was the major contributor in writing the manuscript. All authors read and approved the final manuscript.
